# Multi-kingdom gut microbiota analysis identifies bacterial-viral association in multiple myeloma

**DOI:** 10.3389/fmicb.2026.1798330

**Published:** 2026-05-29

**Authors:** Lu Liu, Jingmei Liu, Jianxia He, Yu Xing, Dai Zhang, Xiaoqing Zhang, Cong Ma, MengYao Xu, Ruiqi Li, Miaoxin Peng, Shuhao Mei

**Affiliations:** 1 Department of Hematology, Xu Chang Central Hospital, Xu Chang, Henan, China; 2 Department of Gastroenterology, Cancer Hospital Affiliated to Shanxi Medical University/Shanxi Province Cancer Hospital/Shanxi Hospital Affiliated to Cancer Hospital, Chinese Academy of Medical Sciences, Taiyuan, Shanxi, China; 3 Department of Hematology, Shanxi Provincial People’s Hospital, Taiyuan, Shanxi, China; 4 Department of Hematology, Nanjing Drum Tower Hospital, Affiliated Hospital of Medical School, Nanjing University, Nanjing, Jiangsu, China

**Keywords:** bacterial-viral association, gut microbiota, gut-brain axis, metagenome sequencing, multiple myeloma

## Abstract

**Introduction:**

Alterations in the gut microbiome are closely associated with the progression of multiple myeloma (MM). Previous research has predominantly focused on the bacterial components of the microbiota; however, the virome, a significant component of the microbiota, also plays a critical role, with bacteriophages influencing bacterial community composition and evolution.

**Methods:**

This study utilized shotgun metagenomic sequencing of fecal samples to explore the interaction between the gut microbiota and MM development. Fecal samples from 28 MM patients and 20 healthy controls were analyzed to evaluate microbial diversity. Taxonomic profiling of both bacterial and viral communities was performed using the Kraken2 classifier.

**Results:**

Our analysis confirmed microbial dysbiosis in MM patients and revealed concomitant changes in both bacterial and viral communities. At the phylum level, this study identified a significant increase in the relative abundance of Pseudomonadota (from 1.63 to 8.88%, *p* < 0.001) and a decrease in Bacillota in MM patients compared to controls. Furthermore, several viral taxa were notably enriched in the MM cohort, including the phylum Heunggongvirae (linear discriminant analysis [LDA] = 4.74, *p* = 0.00003), phylum Uroviricota, and genus *Punavirus* (specifically *Punavirus RCS47*). Functional analysis demonstrated shifts in microbial metabolic pathways associated with MM, including a reduced capacity for amino acid and secondary bile acid biosynthesis and an enrichment of pathways associated with biofilm formation and cationic antimicrobial peptide (CAMP) resistance.

**Discussion:**

This multi-kingdom metagenomic analysis reveals distinct bacterial and viral signatures associated with MM, enhancing our understanding of gut microbial dysbiosis in the disease. These findings lay the groundwork for future mechanistic investigations and highlight the importance of validating these results in larger, independent cohorts.

## 1 Introduction

Multiple myeloma (MM) is a prevalent hematologic malignancy characterized by the infiltration of malignant plasma cells that secrete excessive monoclonal immunoglobulins (Cullis, 2019; Cowan et al., 2018; Jian et al., 2020). With the development of targeted therapy and immunotherapy for MM, the outcomes of patients with MM have improved (Fricker, 2020). Therapeutic resistance remains a substantial clinical challenge, highlighting the need to identify novel biological determinants that guide disease progression.

Recent research has positioned the gut microbiota as a key modulator of cancer pathogenesis (Zheng et al., 2025; Zhu et al., 2024), with its recognition as one of the “Hallmarks of Cancer” emphasizing its essential role in tumor biology (Hanahan, 2022). Microbial communities are believed to influence tumorigenesis through various mechanisms, such as the induction of chronic inflammation, the alteration of cellular proliferation and apoptosis, and the modulation of host immune response (Belkaid and Hand, 2014). Increasing evidence supports the involvement of the gut microbiome in MM development (Jasiński et al., 2022; Calcinotto et al., 2018; Pianko et al., 2019), with MM patients exhibiting notable changes in microbiota composition and diversity (Jian et al., 2020; Zhang et al., 2019).

Bacteria, the predominant microbial component, have been associated with host DNA damage and carcinogenesis (Ahmed et al., 2020). The majority of studies examining the MM-associated microbiome have focused on the bacterial component, primarily using 16S ribosomal RNA (rRNA) sequencing (Rodríguez-García et al., 2024; Zhang et al., 2022). However, the gut hosts a diverse multi-kingdom microbial ecosystem, including viruses, archaea, and fungi. Within this ecosystem, the role of viruses—especially bacteriophages that infect bacteria—remains largely unexplored. Viruses are known to influence bacterial population evolution (Shkoporov et al., 2019; Lozupone et al., 2012). Interestingly, these phages have been shown to regulate microbiota structure and function, contributing to microbiota diversity, stability, and resilience (Shkoporov et al., 2022). Phage-mediated modulation of bacterial populations has garnered increasing interest, particularly in the context of antibiotic resistance (Gogokhia et al., 2019). Furthermore, emerging evidence has suggested that viruses and bacteria may interact synergistically within shared pathological and molecular pathways, potentially amplifying oncogenic signals (Kato et al., 2022). Therefore, a comprehensive characterization of the virome is essential for a more complete understanding of gut ecosystem dysbiosis in MM, even without direct mechanistic validation of bacterial-phage interactions.

The oncogenic potential of viruses and bacteria has been individually explored; however, their potential interactions in the context of cancer, particularly in MM, remain largely uncharacterized. The relationship between shifts in the bacterial community and concurrent alterations in the viral community, along with how these two kingdoms may collectively contribute to the microbial landscape of MM, warrants further investigation. In this study, shotgun metagenomic sequencing was used to simultaneously characterize both the bacteriome and virome in MM patients. By integrating these bacterial and viral components, this study aimed to provide foundational insights into the ecological alterations associated with MM, potentially informing future hypothesis-driven mechanistic and translational studies.

## 2 Materials and methods

### 2.1 Human subject

A total of 48 participants were recruited from Xuchang Central Hospital, consisting of 28 MM patients and 20 healthy controls (Supplementary Table S1). Fecal samples were collected from all participants and immediately stored at −80 °C for further analysis. MM patients met the International Myeloma Working Group diagnostic criteria, which include bone marrow plasma cells exceeding 10%, pathological confirmation of plasma cell tumors, and at least one of the following: anemia caused by the primary disease, bone pain or pathological fractures, renal insufficiency, hypercalcemia, a ratio of involved to uninvolved free light chains greater than 100, or focal lesions detected through magnetic resonance imaging (MRI). To minimize confounding factors that could affect gut microbiome composition, the following exclusion criteria were applied: recent receipt of chemotherapy or radiotherapy. The sample size for the MM group was determined based on the availability of eligible patients during the recruitment period. The control group’s sample size was set at 20 to ensure adequate matching while avoiding unnecessary venipuncture in healthy participants. Research involving humans was approved by the Ethics Committee of the Xuchang Central Hospital (No. 2024–07-004). All participants read and signed an informed consent form.

### 2.2 DNA processing and metagenome profiling

Total DNA was extracted from fecal samples of both MM patients and healthy controls using the MagBeads FastDNA^®^ Kit (MP Biomedicals, Shanghai, China) following the manufacturer’s protocol. DNA concentration and purity were evaluated using a NanoDrop2000 spectrophotometer and a Qubit 2.0 fluorometer (Thermo Fisher, Massachusetts, USA), respectively. Shotgun metagenomic sequencing and subsequent data analysis were conducted by Shaan Probiomicros Co., Ltd. (Xi’an, China) on the BGI platform (MGISEQ-T7, Shenzhen, China). Briefly, raw reads were processed by trimming sequencing adapters and low-quality reads using fastp (v0.23.0). The reads were then aligned with the human genome (GRCh38) (Langmead and Salzberg, 2012) using Bowtie2 software (v2.3.5.1) to remove host contamination.

For taxonomic profiling, high-quality reads were aligned with the MetaPhlAn4 database (Beghini et al., 2021; Truong et al., 2015) to generate relative abundance profiles of microbial communities, including bacteria, archaea, fungi, and viruses, from sequenced metagenomic data. The database comprised 332,371 bacterial genomes, 1,934 archaeal genomes, 18,639 viral genomes, and 3,047 fungal genomes. After filtering human reads, the remaining high-quality non-human reads were classified using Kraken2 against a reference database of all RefSeq bacterial and viral genomes with a confidence threshold of 0.1 (O'Leary et al., 2016). To evaluate the accuracy of viral predictions, true-positive and false-positive classifications were assessed using the R package taxonomizr. Relative abundance was calculated as the proportion of reads classified to each taxon relative to the total number of classified reads. Species abundance and composition were visualized using stacked plots, heatmaps, and pie charts.

### 2.3 Diversity analysis

Alpha diversity indices were calculated using multiple metrics, and statistical comparisons (Shannon index and Simpson index) between the MM and control groups were performed using the Wilcoxon test on R (V2.6–4). Beta diversity, which measures the compositional differences between microbial communities across samples, was assessed using the Bray–Curtis dissimilarity index derived from relative abundance data. A permutational multivariate analysis of variance (PERMANOVA) with 999 permutations was performed to test for differences in community composition between the groups. Results were visualized using the principal coordinate analysis (PCoA) method. These calculations were performed using the “vegan” package in R.

### 2.4 Functional analysis

Species abundance, metabolic pathways, and functional modules were analyzed using HUMAnN3 (Beghini et al., 2021; Franzosa et al., 2018). Functional pathway analysis included metabolic pathway (MetaCyc) and Kyoto Encyclopedia of Genes and Genomes (KEGG) functions. Gut–metabolic modules (GMMs) were used to annotate microbial functional changes. GMMs reflect bacterial metabolism unique to the human intestinal environment, emphasizing anaerobic fermentation processes. GMMs were identified using Bowtie2 (v2.3.5.1).

### 2.5 Microbiome differential analysis and random forest classifier construction

Statistical and machine learning methods were used in this study. Briefly, linear discriminant analysis (LDA) effect size (LEfSe) is a statistical method used to identify key taxa that differ significantly between MM cases and controls. Differential abundance analysis was carried out using LEfSe. Linear discriminant analysis (LDA) was used to estimate microbial abundance differences. Taxa with an LDA score of > 2.0 and a *p*-value of < 0.05 were considered significantly enriched or depleted between the groups.

To evaluate the ability of the gut microbiome to differentiate MM patients from healthy controls, a random forest classifier was constructed using the randomForest R package. All models were constructed to perform a random forest analysis. First, random forest classification models were built based on species abundance data to extract importance scores for each feature (bacterial species and viral species) in differentiating between the case and control groups. The mean decrease in accuracy was used to evaluate the importance of each feature in the model. After obtaining the importance scores, features were sorted in descending order of importance. Subsequently, features were added one by one in descending order of importance to construct prediction models until optimal model performance was achieved.

A stratified 10-fold cross-validation approach was used to evaluate the prediction performance of each feature set, repeated five times to obtain stable estimates. The results from all runs were averaged to produce more objective outcomes. Feature selection and data standardization were performed within each cross-validation fold to prevent data leakage and ensure unbiased performance estimation. With a total sample size of 48, each validation fold contained approximately 4–5 samples. Model performance was assessed using the area under the receiver operating characteristic curve (AUC), with 95% confidence intervals calculated using DeLong’s test. The performance of the final model was evaluated using the “pROC” package to plot the receiver operating characteristic (ROC) curve based on the cross-validation results.

### 2.6 Statistical analysis

Statistical analyses were conducted using R software (R-4.3.1). Demographic and clinical characteristics were compared between groups using Fisher’s exact test for categorical variables, presented as counts and percentages. The Wilcoxon rank-sum test was used to compare the relative abundance of microbial taxa between MM patients and healthy controls. *p*-values were adjusted for multiple comparisons using the Benjamini–Hochberg procedure, with a false discovery rate (FDR) of < 0.05 considered statistically significant. For LEfSe analysis, taxa with an LDA score > 2.0 and all *p*-values of < 0.05 were considered statistically significant. We corrected for multiple testing to control the false discovery rate using the Benjamini–Hochberg procedure and used a significance threshold of 0.01 on adjusted p-values.

## 3 Results

### 3.1 Diversity and composition of gut bacteriome and virome in patients with MM and healthy controls

**Figure 1:**
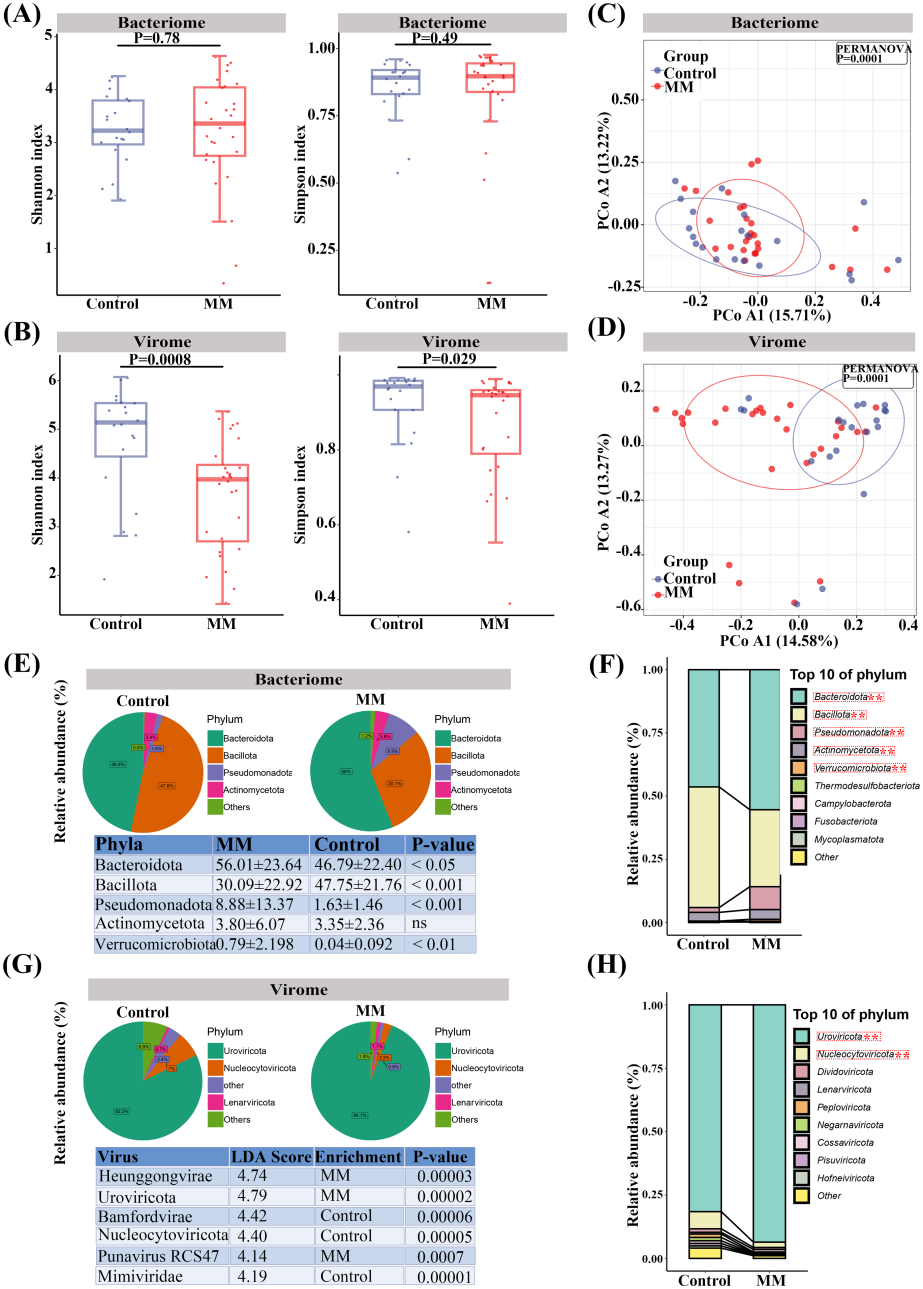
Composition of the microbial community in MM patients and healthy controls based on metagenomic data. **(A,B)** Alpha diversity (Shannon index and Simpson index) for bacterial and viral species in both the control and MM groups, presented as box plots with median, interquartile range, minimum, and maximum values. **(C,D)** Analysis of microbiota community structure in gut samples from the control and MM groups based on principal coordinate analysis (PCoA) of the Bray–Curtis dissimilarity as assessed by the PERMANOVA. **(E,G)** Proportions and comparisons of bacterial and viral abundance at the phylum level between the control and MM groups. **(F,H)** Stacked bar chart depicting bacterial and viral compositions at the phylum level.

We assessed the gut microbial composition in patients with MM (n = 28) and healthy controls (n = 20) using shotgun metagenomic sequencing. Alpha and beta diversity analyses were performed to assess the richness and diversity of bacterial and viral species. The alpha diversity was evaluated by calculating the Shannon and Simpson indices. At the bacterial level, no significant differences in alpha diversity were observed between the MM and control groups (Shannon index: *p* = 0.78; Simpson index: *p* = 0.49; Figure 1A). In contrast, viral alpha diversity was significantly lower in MM patients than in healthy controls, with reductions in both the Shannon index (*p* = 0.008) and the Simpson index (*p* = 0.029) (Figure 1B). Beta diversity analysis based on the Bray–Curtis dissimilarity index revealed distinct clustering of microbial communities between the two groups. PCoA further demonstrated significant differences in both bacterial (PERMANOVA, *p* = 0.0001; Figure 1C) and viral (PERMANOVA, *p* = 0.0004; Figure 1D) community compositions between MM patients and healthy controls.

The relative abundance of bacterial and viral taxa at multiple taxonomic levels was compared between the MM and control groups (Figures 1E,G). At the phylum level, the gut microbiota of both groups was dominated by Bacillota (30.1%) and Bacteroidota (56.0%). Compared to healthy controls, MM patients exhibited a reduced relative abundance of Bacillota (*p* < 0.001) and an increase in the abundance of Bacteroidota (*p* < 0.05) and Pseudomonadota (*p* < 0.001) (Figure 1E). Among viral taxa, the phylum Uroviricota comprised 94.1% of the viral sequences. Several viral taxa were differentially abundant between the groups, including enrichment of the phyla Heunggongvirae and Uroviricota, as well as the species *Punavirus RCS47*, in MM patients (Figure 1G).

At lower taxonomic levels, a reduced relative abundance of the species *Anaerostipes hadrus* was observed in the MM group compared to the control group (Figure 1F). At the family level, MM patients showed a decrease in the abundance of Lachnospiraceae (LDA = 4.92) and an increase in Enterobacteriaceae (LDA = 4.55). At the genus level, patients with MM exhibited reduced abundances of *Roseburia* (LDA = 4.02) and *Blautia* (LDA = 4.51), both of which are known to produce short-chain fatty acids (SCFAs) with anti-inflammatory properties. However, the genera *Alistipes* (LDA = 4.06) and *Bacteroides caccae* (LDA = 3.84) exhibited higher relative abundances in MM (Supplementary Figure S1A).

Regarding viral community, MM patients showed a significant increase in the abundance of phylum Uroviricota (LDA = 4.79) and a decrease in the abundance of phylum Nucleocytoviricota (LDA = 4.40) (Figure 1H). In addition, the family Mimiviridae (LDA = 4.19) and the species *Punavirus RCS47* (LDA = 4.14) were significantly reduced in the MM group compared to the control group (Supplementary Figure S1B). In both the control and MM groups, the composition of bacteria and viruses was relatively stable across dynamic points at the phylum level, whereas the composition and relative abundance were more variable across dynamic points at the species level (Supplementary Figures S1C,D), suggesting that taxonomic resolution influences the detection of microbial alterations associated with MM. Volcano plots identified the differentially abundant gut bacterial and viral species between the control and MM patients at the species level, including both significantly enriched and depleted species in the MM group (Supplementary Figures S2A,B). The differentially abundant taxa between the control and MM groups were identified (Supplementary Figure S2C). These results demonstrate notable differences in the abundance of various bacterial and viral taxa between MM patients and healthy controls, marked by shifts in both dominant and less abundant species.

### 3.2 Differential bacterial and viral taxa between MM patients and healthy controls

**Figure 2:**
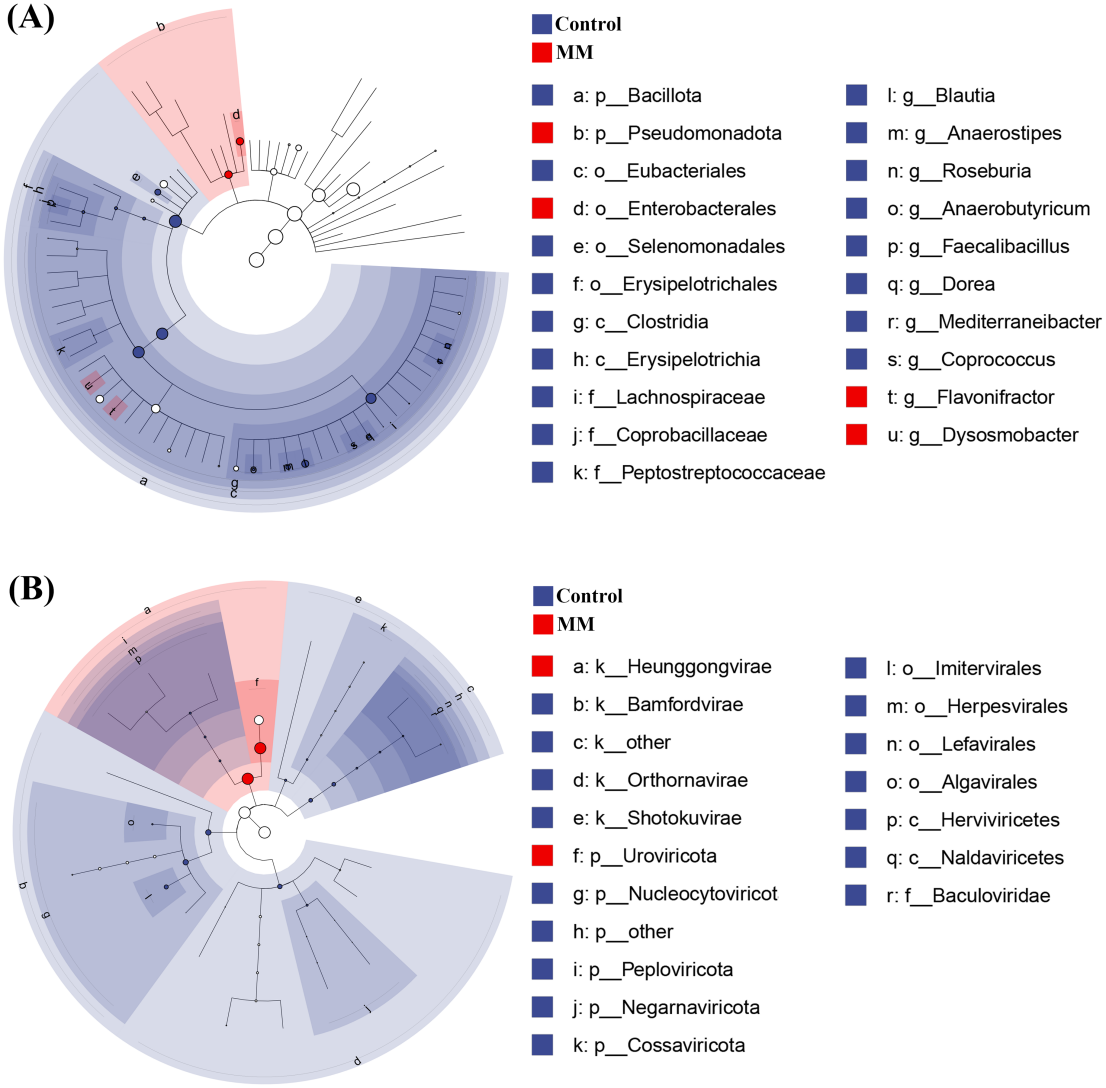
Differential bacterial and viral taxa across populations. **(A)** Linear discrimination analysis of effect size (LEfSe) cladograms highlighting differences in bacterial taxa between the control and MM groups. **(B)** Linear discrimination analysis of effect size (LEfSe) cladograms illustrating differences in the viral taxa between the control and MM groups.

To identify specific microbial taxa associated with MM, LEfSe was performed to compare relative abundances between the two groups at various taxonomic levels (Figure 2A). In the control group, the phylum Bacillota (*p* < 0.01) and the family Lachnospiraceae (*p* < 0.001) were identified as characteristic bacterial taxa. In contrast, MM patients exhibited significant enrichment of the phylum Pseudomonadota (*p* < 0.05) and the family Enterobacteriaceae (*p* < 0.05) (Supplementary Figures S3A,B).

For the viral community, we constructed a phylogenetic tree based on viral abundance profiles (Figure 2B). LEfSe analysis revealed that the phylum Nucleocytoviricota (*p* < 0.001) and the family Mimiviridae (*p* < 0.001) were characteristic of the control group. In MM patients, the order Crassvirales (*p* < 0.01), the phylum Uroviricota (*p* < 0.001), and the species *Punavirus RCS47* (*p* < 0.001) were significantly enriched (Supplementary Figure S3C).

### 3.3 Co-occurrence network analysis of bacterial and viral communities in MM

**Figure 3:**
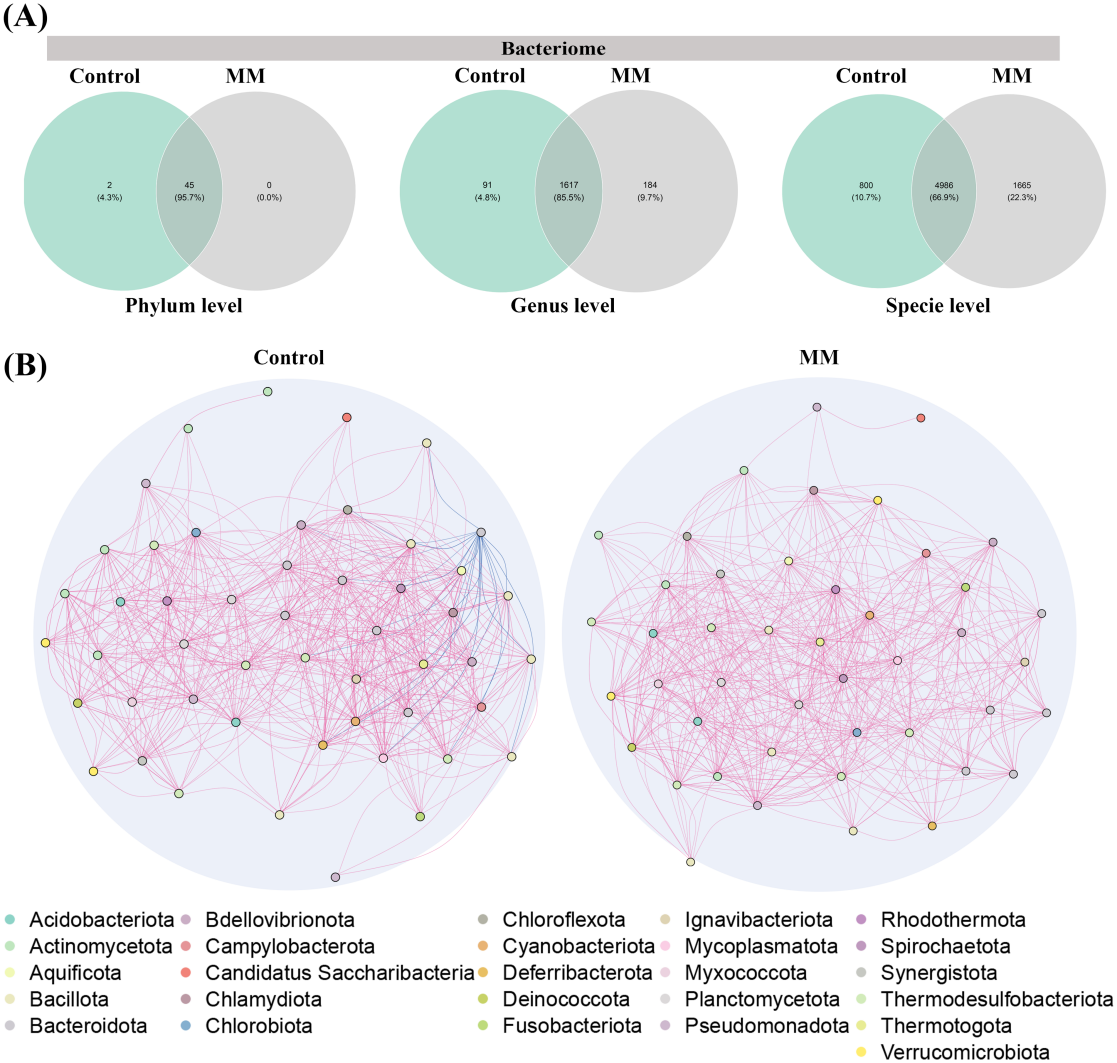
Co-occurrence network analysis among bacterial taxa in MM patients. **(A)** Venn diagram depicting bacterial variations at the phylum, genus, and species levels between the control and MM groups. **(B)** Co-occurrence networks for viral taxa between the control and MM groups. The nodes are colored according to phylum and weighted by the degree of each species.

Co-occurrence network analysis was performed to explore the interrelationships within microbial communities, focusing on both bacterial and viral taxa in MM patients and healthy controls. Venn diagram analysis revealed the distribution of shared and unique taxa between the groups (Figure 3A). At the phylum level, 47 bacterial phyla were detected, with 2 unique to the control group. At the genus level, 1,617 genera were shared between the groups, while 184 genera were unique to MM patients and 91 genera were unique to controls. At the species level, 7,986 species were shared, with 1,665 species unique to MM and 800 species unique to controls. The co-occurrence network constructed for the bacterial community in the MM group consisted of 45 nodes (representing taxa) and 521 edges (representing significant correlations between taxa) (Figure 3B). The network topology was characterized by an average path length of 1.531, a clustering coefficient of 0.72, and a modularity index of 0.186.

**Figure 4:**
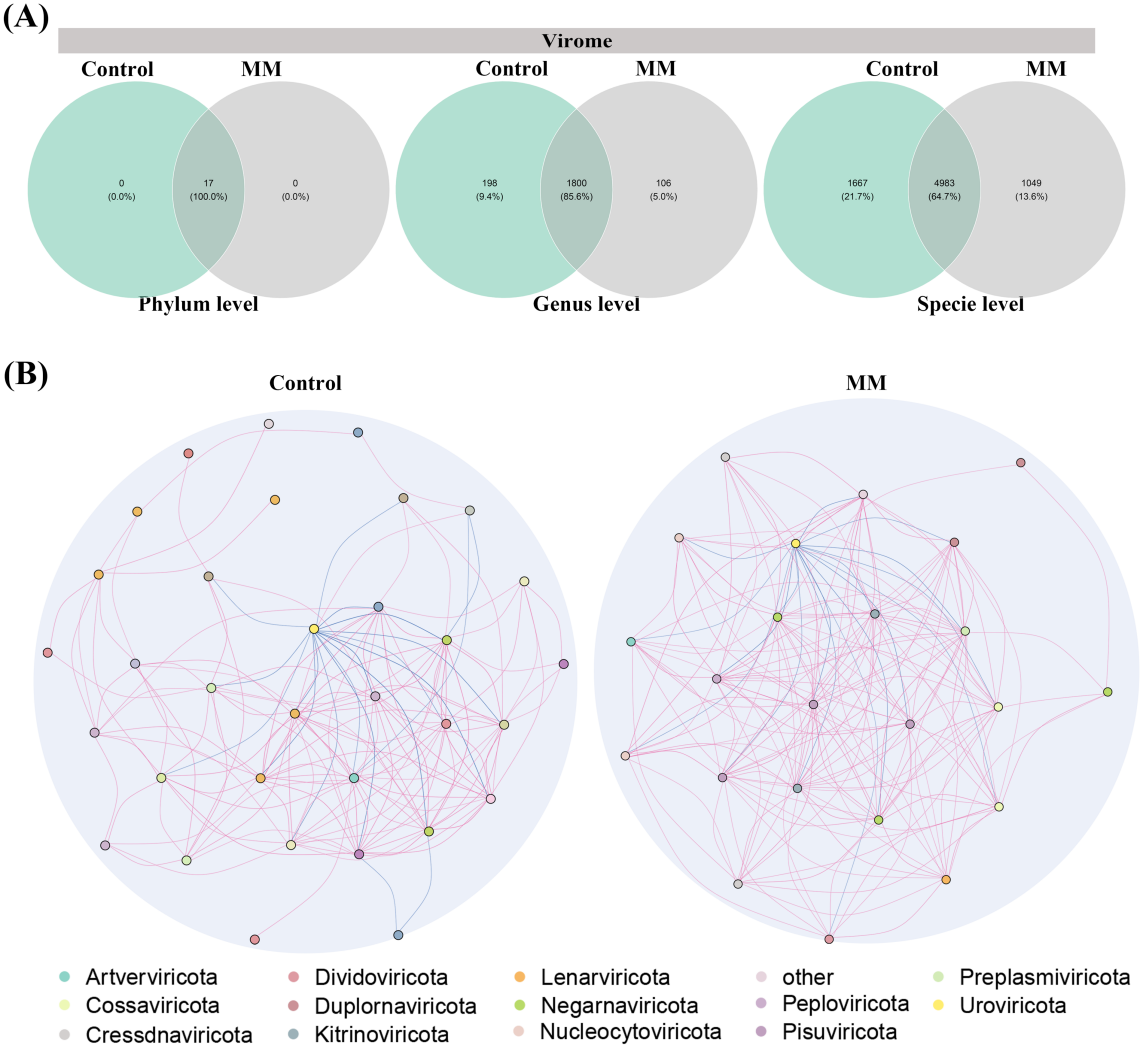
Co-occurrence network analysis among viral taxa in MM patients. **(A)** Venn diagram depicting viral variations at the phylum, genus, and species levels between the control and MM groups. **(B)** Co-occurrence networks for viral taxa in the control and MM groups. The nodes in the network are colored according to phylum and weighted by the node degree of each species.

For the viral community, Venn diagram analysis identified 17 phyla in total. At the genus level, 1,800 genera were shared between the groups, and at the species level, 4,983 species were shared (Figure 4A). The co-occurrence network for the viral community in the MM group comprised 23 nodes and 167 edges (Figure 4B).

### 3.4 Multi-kingdom co-abundance network analysis in patients with MM

**Figure 5:**
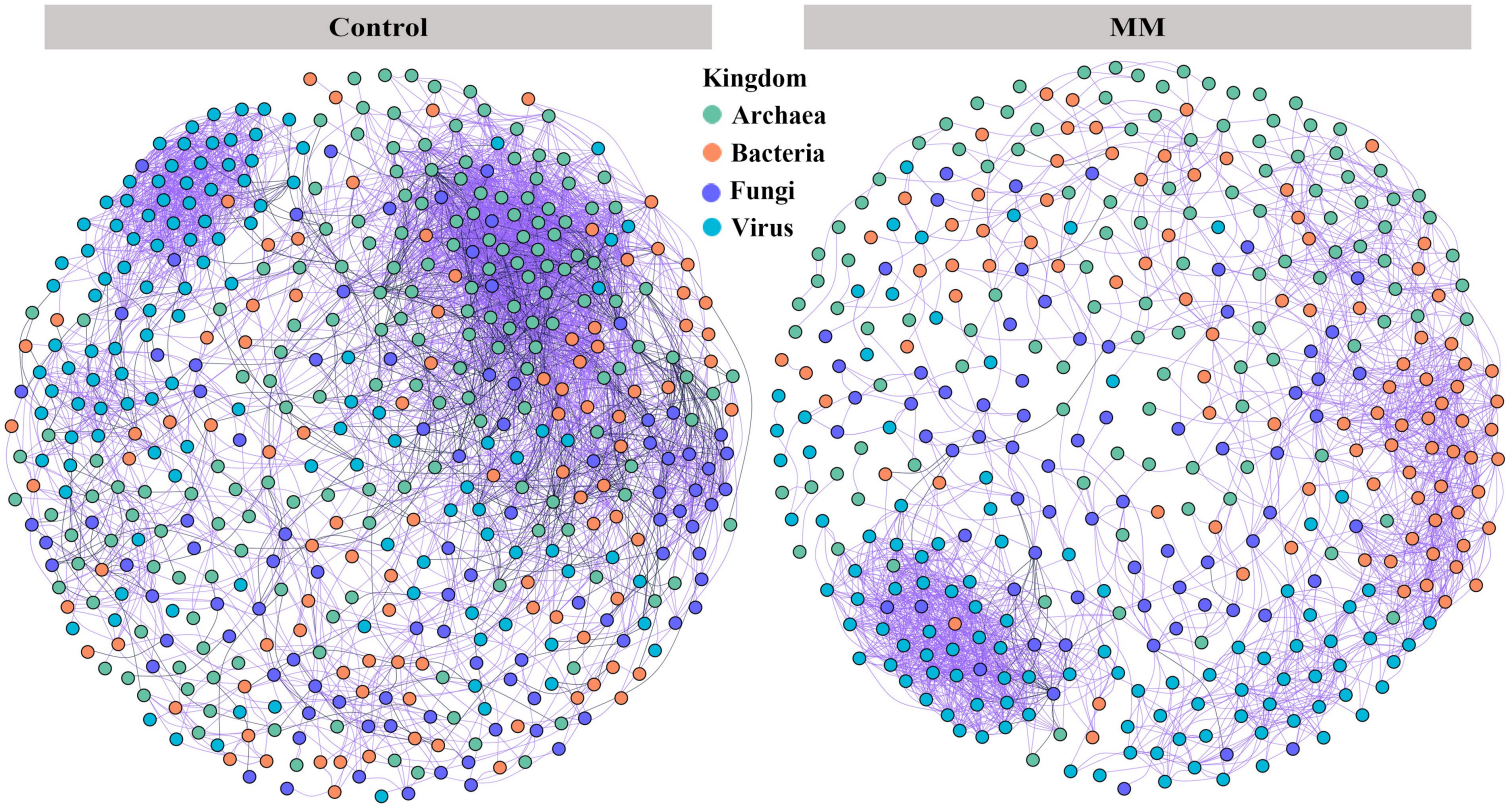
Co-abundance associations among multiomics taxa in MM patients. Co-abundance networks in the control and MM groups, showing correlations of above 0.7, with a *p*-value of < 0.05.

To investigate potential associations across microbial kingdoms, a multi-kingdom co-abundance network analysis was performed, incorporating differentially abundant taxa from bacteria, viruses, archaea, and fungi (Figure 5). After adjusting for association coefficients (r > 0.7), the resulting network revealed a more complex structure in the MM group than in the control group, with a higher number of significant co-abundance associations among taxa. Notably, multiple co-abundance associations between bacterial and viral taxa were identified, indicating potential ecological linkages between these kingdoms in the context of MM.

### 3.5 Classification performance of bacterial and viral signatures for MM

To assess the potential of microbial signatures in identifying MM status, a random forest classifier was used, training the model on species-level taxonomic profiles of the microbiomes. The models trained on bacterial and viral signatures demonstrated high discriminatory performance, with an area under the receiver operator characteristic (ROC) curve of 0.9661 (95% CI: 0.913) and 0.9946 (95% CI: 0.982), respectively (Supplementary Figure S4A). The results of the random forest analysis identified bacterial taxa, including *Anaerostipes hadrus* and *Blautia luti* (Supplementary Figure S4B). These taxa represent promising candidate biomarkers for MM, warranting further exploration in mechanistic studies.

### 3.6 Functional alterations in the gut microbiota in patients with MM

To investigate the potential functional implications of the observed microbial shifts, functional profiling of the metagenomic data was performed using KEGG Orthology (KO) annotations, KEGG modules, and pathway-level analyses.

**Figure 6:**
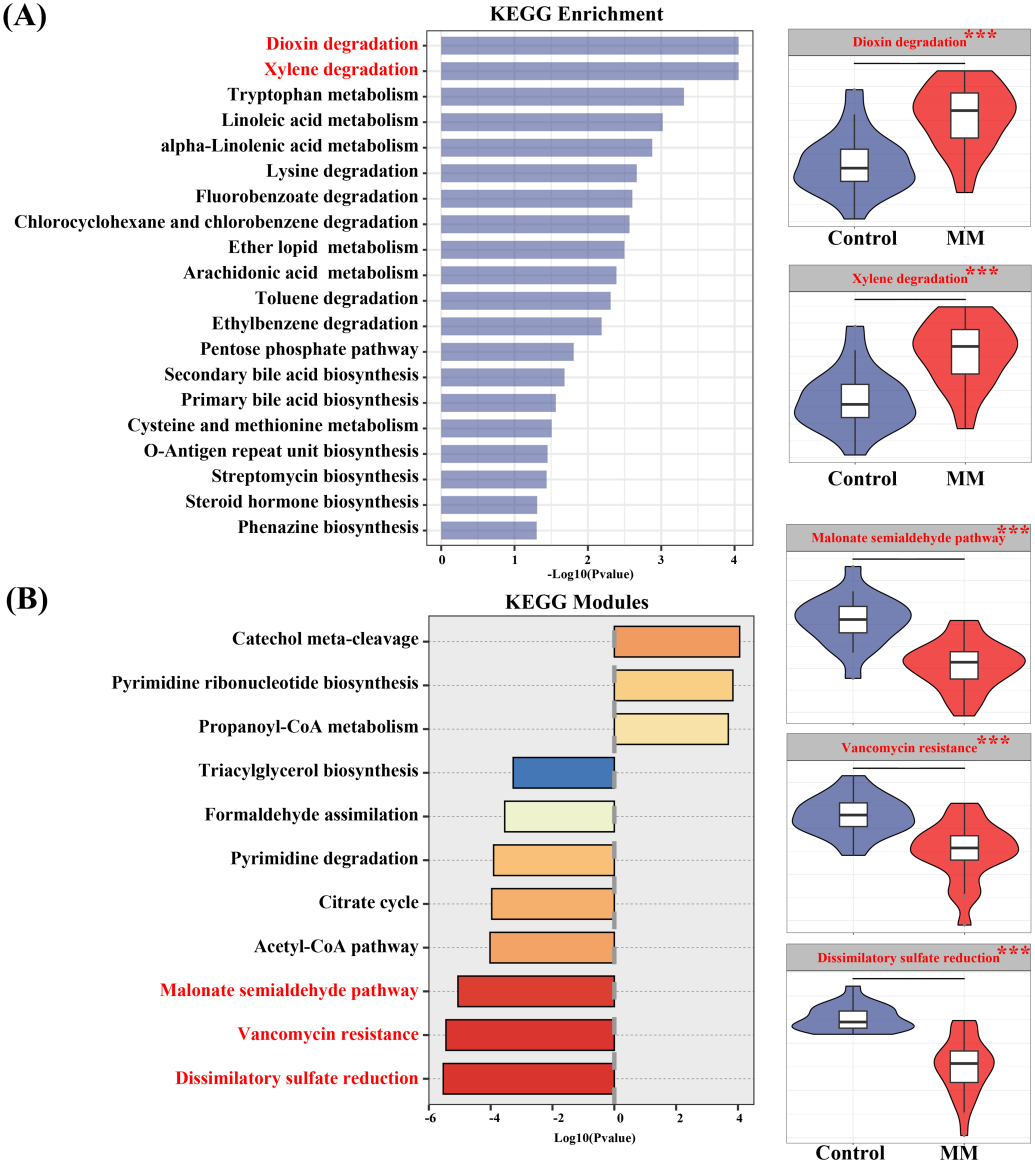
Functional alterations in gut microbiota in MM patients. Functional annotation of differential microbiota based on KEGG pathways **(A)** and KEGG modules **(B)**.

As shown in Figure 6A, pathway-enrichment analysis revealed that KOs involved in the degradation of dioxin and xylene were significantly more abundant in the MM group compared to the control group. At the KEGG module level, the MM group exhibited significant enrichment of modules related to catechol meta-cleavage, pyrimidine ribonucleotide biosynthesis, and propanoyl-CoA metabolism. Conversely, modules associated with the malonate semialdehyde pathway, vancomycin resistance, and dissimilatory sulfate reduction were significantly depleted in MM patients (Figure 6B).

Further analysis of functional pathways identified an enrichment of genes associated with biofilm formation, including those from *Escherichia coli* and *Vibrio cholerae*, and cationic antimicrobial peptide (CAMP) resistance in the MM group (Supplementary Figure S5A). These pathways involving *Escherichia coli* biofilm formation and CAMP resistance played important roles (Supplementary Figures S6, S7). Network analysis linking gut microbes to genes involved in *Escherichia coli* and CAMP resistance (Supplementary Figure S5B, left). In the biofilm formation of *Escherichia coli*, the gene glgC (K00975) exhibited reduced abundance in MM patients. In the CAMP resistance pathway, htrA (K04771) was significantly depleted. Notably, the genes involved in *Escherichia coli*, which exhibited reduced abundance in MM patients, were significantly elevated, such as rpoS (K03087), oxyR (K04761), mlrA (K21089), and adrB (K21090). In addition, eptA (K03760), pmrK (K07264), tolC (K12340), and sapF (K19230) were elevated in the CAMP resistance pathway (Supplementary Figure S5B, right).

### 3.7 GBM and GMM analysis of gut microbiota in patients with MM

**Figure 7:**
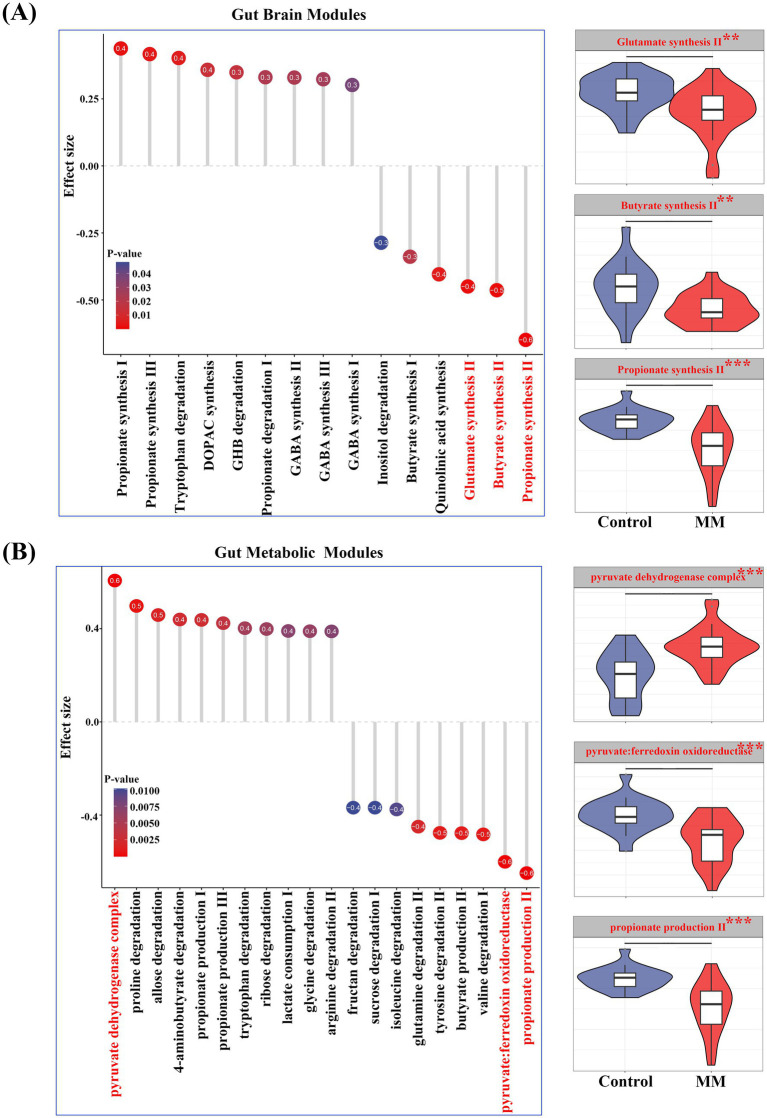
Gut–brain and gut–metabolic modules **(A,B)**. Functional carriage of the gut–brain module (GBM) and gut–metabolic module (GMM).

To evaluate the functional potential of the gut microbiota in MM, the abundance of gut–brain modules (GBMs) and gut–metabolic modules (GMMs) was characterized (Valles-Colomer et al., 2019; Vieira-Silva et al., 2016). A total of 15 GBMs and 20 GMMs were identified in the dataset (Figures 7A,B). Among the GBMs, modules associated with the synthesis of amino acids and short-chain fatty acids—including glutamate, butyrate, and propionate—showed decreased abundance in the MM group compared to the control group (Figure 7A). For the GMMs, the pyruvate dehydrogenase complex was enriched in the MM group; pyruvate ferredoxin oxidoreductase and propionate production were predominant in the control group (Figure 7B).

## 4 Discussion

Accumulating evidence has highlighted the presence of a low-abundance yet functionally significant gut microbiome, which plays a pivotal role in human health and disease (Zheng et al., 2025). In the current study, shotgun metagenomic sequencing was used to characterize the multi-kingdom gut microbiota, including both bacterial and viral communities, in patients with MM. Our analysis revealed significant alterations in both the bacteriome and virome of MM patients compared to healthy controls. Notably, significant differences in beta diversity were observed for both bacterial and viral communities between the groups, indicating that shifts in microbial community structure may be associated with MM. These findings provide a comprehensive description of the bacterial, viral, and other microbial communities in the guts of MM patients. By simultaneously characterizing both kingdoms, this study broadens our understanding of gut microbial dysbiosis in MM and proposes potential inter-kingdom associations that warrant further investigation. We appreciate that clinical trials are inherently different from metagenomic studies, which are often exploratory in nature. Given the exploratory nature of this study and the relatively modest sample size, although statistically significant, the association observed in the overall study was not definitive. We have less power to detect this association in our smaller subset compared with the larger studied cohort. It has been demonstrated that microbiome association studies with small sample sizes tend to overestimate effect sizes and suffer from insufficient statistical power, increasing the risks of both false-positive and false-negative discoveries (Kers and Saccenti, 2021). Although limited in power by the small sample size, we aimed to integrate the importance of the variables studied.

In healthy individuals, microbial translocation to the gut from other body sites is sporadic and transient (Tan et al., 2023). In the present study, beneficial bacterial taxa within the phylum Bacillota were predominantly enriched in healthy controls. In contrast, MM patients, with compromised immune systems, appear more susceptible to specific microbial alterations (Rossi et al., 2021). A significant decrease in the abundance of Bacillota was found in MM patients, suggesting a shift in the gut microbial composition associated with the disease.

A prominent feature of the bacterial composition in MM patients was a significant increase in the phylum Pseudomonadota (from 1.63 to 8.88%, representing a 4.45-fold increase). Similar increases have been reported in colorectal cancer patients (21.85%) and are associated with metastatic stages (Ma et al., 2025). This phylum includes various opportunistic pathogens that proliferate in inflammatory diseases (Leão et al., 2023) and harbor genes associated with antibiotic resistance (Gomes et al., 2025). A total of 1,231 differentially abundant taxonomic units were identified within the bacterial domain. Among these units, the family Enterobacteriaceae was significantly enriched in MM patients. This family comprises opportunistic pathogens—such as *Escherichia coli*, *Klebsiella pneumoniae*, and *Salmonella* species—often associated with gastrointestinal infections (De Angelis et al., 2020). Enrichment of Enterobacteriaceae has also been noted in other MM patient cohorts (Yang et al., 2025). MM patients with weakened immune function are more prone to systemic infections, such as bacteremia and sepsis, and the presence of these opportunistic pathogens in the gut may contribute to this increased susceptibility (Satheeshkumar et al., 2023), although this hypothesis requires further direct investigation. Additionally, *Flavonifractor* and *Dysosmobacter*, both of which are associated with metabolic disorders, were observed in MM patients (Li et al., 2025; Le Roy et al., 2022). While SCFA concentrations were not directly measured, the depletion of key butyrate producers such as *Roseburia* and *Blautia* suggests that the profile of SCFAs available to the host may be altered in MM patients. Given the established anti-inflammatory properties of these bacteria (Arnoldini et al., 2025), their reduced abundance could impact gut barrier integrity and immune regulation in MM.

Within the viral community, several bacteriophage taxa were notably enriched. Bacteriophages play a critical role in shaping bacterial community composition and evolution (Reyes et al., 2013). A significant increase in the phage phylum Heunggongvirae (tail bacteriophages) corresponded to an increase in Pseudomonadota, suggesting potential associations between these viral and bacterial taxa. Bacteriophages may proliferate in parallel with their bacterial hosts, reflecting co-occurrence dynamics between bacteria and viruses (Shuwen and Kefeng, 2022). Phage-mediated horizontal gene transfer can facilitate the spread of antibiotic resistance genes and virulence factors, and phage-induced bacterial lysis may release endotoxins that exacerbate inflammation (Gogokhia et al., 2019). CrAss-like phages and the family Mimiviridae were prominent in the control group. CrAss-like phages, among the most abundant bacteriophages in the human gut, have been associated with maintaining ecological balance (Guerin et al., 2018). Mimiviridae, large DNA viruses with genomes reaching up to 1.5 Mb, encode various metabolic enzymes and tRNAs (Claverie and Abergel, 2018). These giant viruses interact with mammalian immune cells and may influence immune regulation (Kalafati et al., 2022). The presence of Mimiviridae in the gut of healthy individuals suggests a possible symbiotic or co-evolutionary relationship. Previous studies have shown correlations between bacteriophages and bacteria in cancer contexts (Ritz et al., 2024; Liu et al., 2025), and phage–host associations may directly or indirectly affect bacterial proliferation (Shkoporov et al., 2022; Reyes et al., 2013). These observations suggest that distinct bacteriophage communities may be present in MM patients, which warrants further investigation.

The functional profile of the gut microbiome in MM patients exhibited an imbalanced pattern of synthesis capabilities and resistance mechanisms. Pathways involved in amino acid and vitamin synthesis were downregulated in MM patients, potentially impacting the nutritional support available to both the host and the microbial community (Yang et al., 2023). In addition, the phosphotransferase system (PTS) and glycolysis pathways were inhibited in MM patients. The PTS system plays a role in bacterial adaptation to stress and antibiotic resistance (Jiang et al., 2023). Biofilm formation and CAMP resistance were enriched in MM samples, which may reflect bacterial adaptations that enhance survival under adverse conditions. Secondary bile acids, such as deoxycholic acid (DCA) and lithocholic acid (LCA), are important immune regulatory molecules influencing immune responses (van der Lugt et al., 2022). Accumulation of bile acids and bile acid-derived metabolites has been implicated in triggering inflammation and tumorigenesis (Cai et al., 2022). Moreover, bile acids are involved in lipid, glucose, and energy metabolism (Jia et al., 2021). Our findings indicated that the secondary bile acid biosynthesis pathway was significantly downregulated in MM patients, suggesting that alterations in bile acid metabolism may contribute to metabolic abnormalities in MM patients. Overall, these functional alterations suggest a shift in the gut microbiome of MM patients toward a configuration that may favor pathogenic potential, although direct mechanistic links remain to be elucidated.

## 5 Limitations

Several limitations of this study should be acknowledged. The small sample size (28 MM patients and 20 controls) restricts the generalizability of the findings. Expanding the sample size would enhance model precision and possibly unveil additional significant associations. Additionally, the cross-sectional design limits the ability to draw causal conclusions regarding the relationship between gut microbial alterations and MM pathogenesis. Longitudinal studies are required to determine whether the observed changes precede or follow disease onset and if they correlate with clinical outcomes. While this study demonstrates that bacterial and viral species can distinguish MM patients from healthy controls, the functional and mechanistic basis of potential inter-kingdom relationships remains unexplored. Specific interactions between gut microbes and their host, along with the underlying molecular mechanisms, require further investigation in cell-based and animal models. The upper bound of the 95% confidence interval for the AUC reached 1.000, which may be attributed to the limited sample size and should be interpreted with caution. Validation in larger independent cohorts is necessary to obtain more precise performance estimates. Nonetheless, this study provides a foundational characterization of the multi-kingdom gut microbiome in MM and generates hypotheses for future mechanistic and translational investigations.

## 6 Conclusion

In summary, this analysis demonstrated both hypothesized and unexpected interactions, and we characterized the multi-kingdom gut microbiota, including bacterial and viral communities, in MM patients. The findings reveal distinct alterations in both bacterial and viral taxa associated with MM, marked by a decrease in potentially beneficial bacteria and an enrichment of opportunistic pathogens and associated bacteriophages. These results expand our understanding of gut microbial dysbiosis in MM and highlight potential inter-kingdom associations that warrant further exploration. Future studies integrating mechanistic experiments and validation in independent cohorts will be critical to determine the clinical and biological relevance of these findings.

## Data Availability

The datasets presented in this study can be found in online repositories. The names of the repository/repositories and accession number(s) can be found in the article/Supplementary material.

## Supplementary material

SUPPLEMENTARY FIGURE S1 **(A,B)** Stacked bar-chart showing bacterial and viral compositions at the family, genus, and species levels. **(C,D)** Dynamic gut microbiome composition at the phylum and species levels.

SUPPLEMENTARY FIGURE S2 **(A,B)** Volcano plot showing correlations between p-values and relative intensities (fold change, FC) for representative gut bacteria and viruses in Control and MM. Red and blue dots indicate significant differences. **(C)** Histogram of LDA scores computed for differentially abundant taxa between Control and MM groups.

SUPPLEMENTARY FIGURE S3 **(A,B)** Relative abundance of the top 10 significant differential bacteria compared between Control and MM groups using the Metastats test. **(C)** Relative abundance of the top five significant differential viruses was compared between Control and MM groups using the Metastats test.

SUPPLEMENTARY FIGURE S4 Classification of MM status by the abundances of gut signatures **(A)** ROC analysis for classifying of MM status using gut bacterial and viral signatures. **(B)** Random forest analysis showing the importance scores for bacteria between Control and MM groups.

SUPPLEMENTARY FIGURE S5 Microbial functional alterations among MM patients. **(A)** Differentially enriched KEGG orthology (KO) genes between MM patients and controls. **(B)** Kyoto Encyclopedia of Genes and Genomes (KEGG) orthology (KO) genes associated with *Escherichia coli* and CAMP resistance (m02026 and m01503) are shown (left). Box plots show the relative abundance of the five most enriched KO genes in MM (right).

SUPPLEMENTARY FIGURE S6 KEGG pathway diagram of biofilm formation in *Escherichia coli*.

SUPPLEMENTARY FIGURE S7 KEGG pathway diagram of cationic antimicrobial peptide (CAMP) resistance.
