# Correction: Multi-kingdom gut microbiota analysis identifies bacterial-viral association in multiple myeloma

**DOI:** 10.3389/fmicb.2026.1927101

**Published:** 2026-07-23

**Authors:** Lu Liu, Jingmei Liu, Jianxia He, Yu Xing, Dai Zhang, Xiaoqing Zhang, Cong Ma, MengYao Xu, Ruiqi Li, Miaoxin Peng, Shuhao Mei

**Affiliations:** 1 Department of Hematology, XuChang Central Hospital, Xu Chang, Henan, China; 2 Department of Gastroenterology, Cancer Hospital Affiliated to Shanxi Medical University/Shanxi Province Cancer Hospital/Shanxi Hospital Affiliated to Cancer Hospital, Chinese Academy of Medical Sciences, Taiyuan, Shanxi, China; 3 Department of Hematology, Shanxi Provincial People's Hospital, Taiyuan, Shanxi, China; 4 Department of Hematology, Nanjing Drum Tower Hospital, Affiliated Hospital of Medical School, Nanjing University, Nanjing, Jiangsu, China

**Keywords:** bacterial-viral association, gut microbiota, gut-brain axis, metagenome sequencing, multiple myeloma

In the published article, there was an error in the affiliations for three co-authors. During the proof stage, the authors inadvertently failed to update the affiliations for Jingmei Liu, Jianxia He, and Miaoxin Peng. The author order remains unchanged.

For author Jingmei Liu (co-first author):

Affiliation “Department of Hematology, Xu Chang Central Hospital, Xu Chang, Henan, China” was erroneously included in the paper. This affiliation has now been removed. Affiliation “Department of Gastroenterology, Cancer Hospital Affiliated to Shanxi Medical University/Shanxi Province Cancer Hospital/Shanxi Hospital Affiliated to Cancer Hospital, Chinese Academy of Medical Sciences, Taiyuan, Shanxi, China” was omitted from the paper. This affiliation has now been added.

For author Jianxia He (co-first author):

Affiliation “Department of Hematology, XuChang Central Hospital, Xu Chang, Henan, China” was erroneously included in the paper. This affiliation has now been removed. Affiliation “Department of Hematology, Shanxi Provincial People's Hospital, Taiyuan, Shanxi, China” was omitted from the paper. This affiliation has now been added.

For author Miaoxin Peng:

Affiliation “Department of Hematology, XuChang Central Hospital, Xu Chang, Henan, China” was erroneously included in the paper. This affiliation has now been removed. Affiliation “Department of Hematology, Nanjing Drum Tower Hospital, Affiliated Hospital of Medical School, Nanjing University, Nanjing, Jiangsu, China” was omitted from the paper. This affiliation has now been added.

The corrected author list and affiliations are:

Lu Liu^1^, Jingmei Liu^2^, Jianxia He^3^, Yu Xing^1^, Dai Zhang^1^, Xiaoqing Zhang^1^, Cong Ma^1^, MengYao Xu^1^, Ruiqi Li^1^, Miaoxin Peng4, Shuhao Mei^1^

^1^Department of Hematology, Xu Chang Central Hospital, Xu Chang, Henan, China

^2^Department of Gastroenterology, Cancer Hospital Affiliated to Shanxi Medical University/Shanxi Province Cancer Hospital/Shanxi Hospital Affiliated to Cancer Hospital, Chinese Academy of Medical Sciences, Taiyuan, Shanxi, China

^3^Department of Hematology, Shanxi Provincial People's Hospital, Taiyuan, Shanxi, China

^4^Department of Hematology, Nanjing Drum Tower Hospital, Affiliated Hospital of Medical School, Nanjing University, Nanjing, Jiangsu, China

The original version of this article has been updated.

