# Supplementary figures and images for: Multi-kingdom gut microbiota analysis identifies bacterial-viral association in multiple myeloma
Source: Front Microbiol. 2026 May 29;17:1798330. doi: 10.3389/fmicb.2026.1798330 (PMC13262065; doi:10.3389/fmicb.2026.1798330)

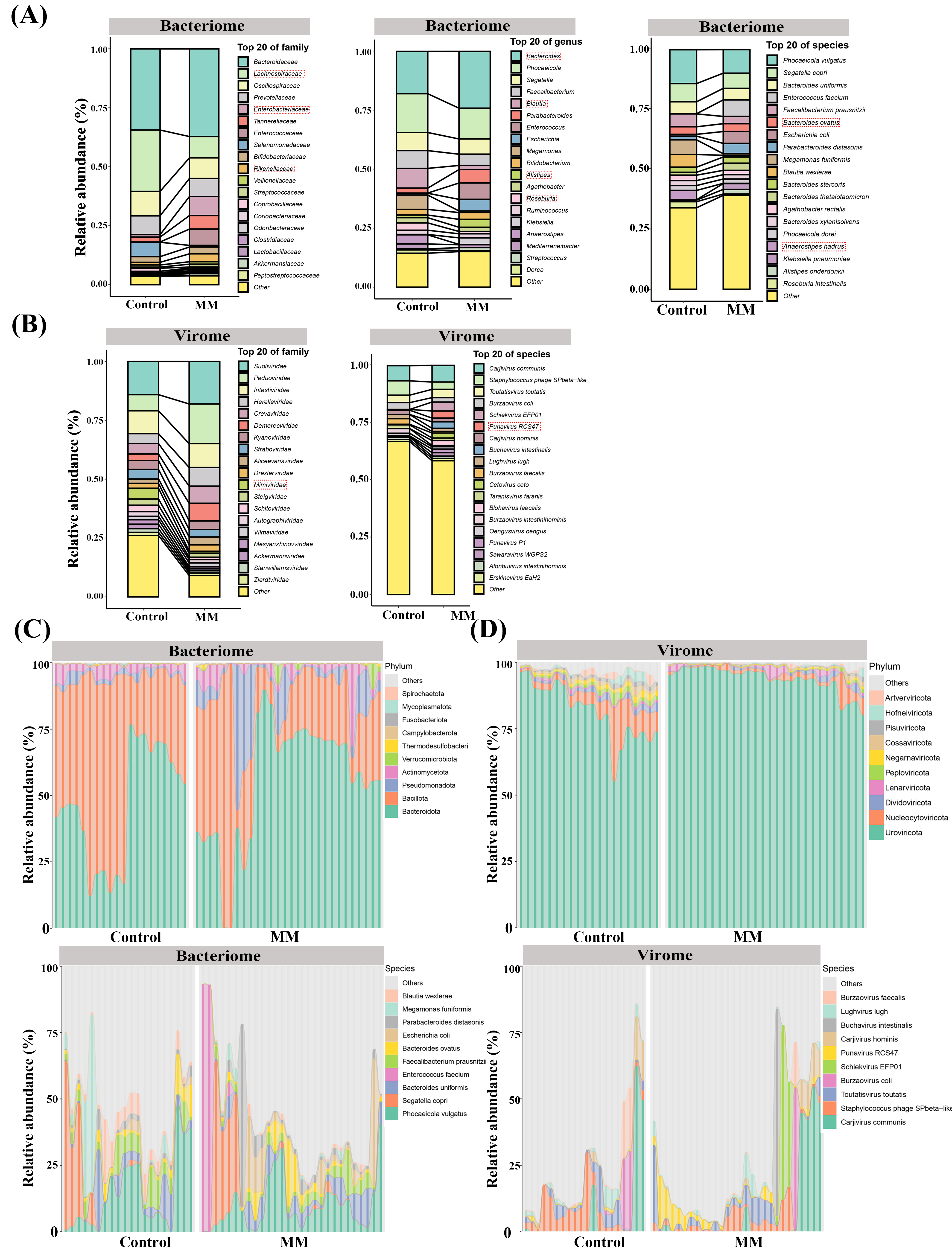

Supplement: SUPPLEMENTARY FIGURE S1 — (A,B) Stacked bar-chart showing bacterial and viral compositions at the family, genus, and species levels. (C,D) Dynamic gut microbiome composition at the phylum and species levels. [file Image_1.tif]

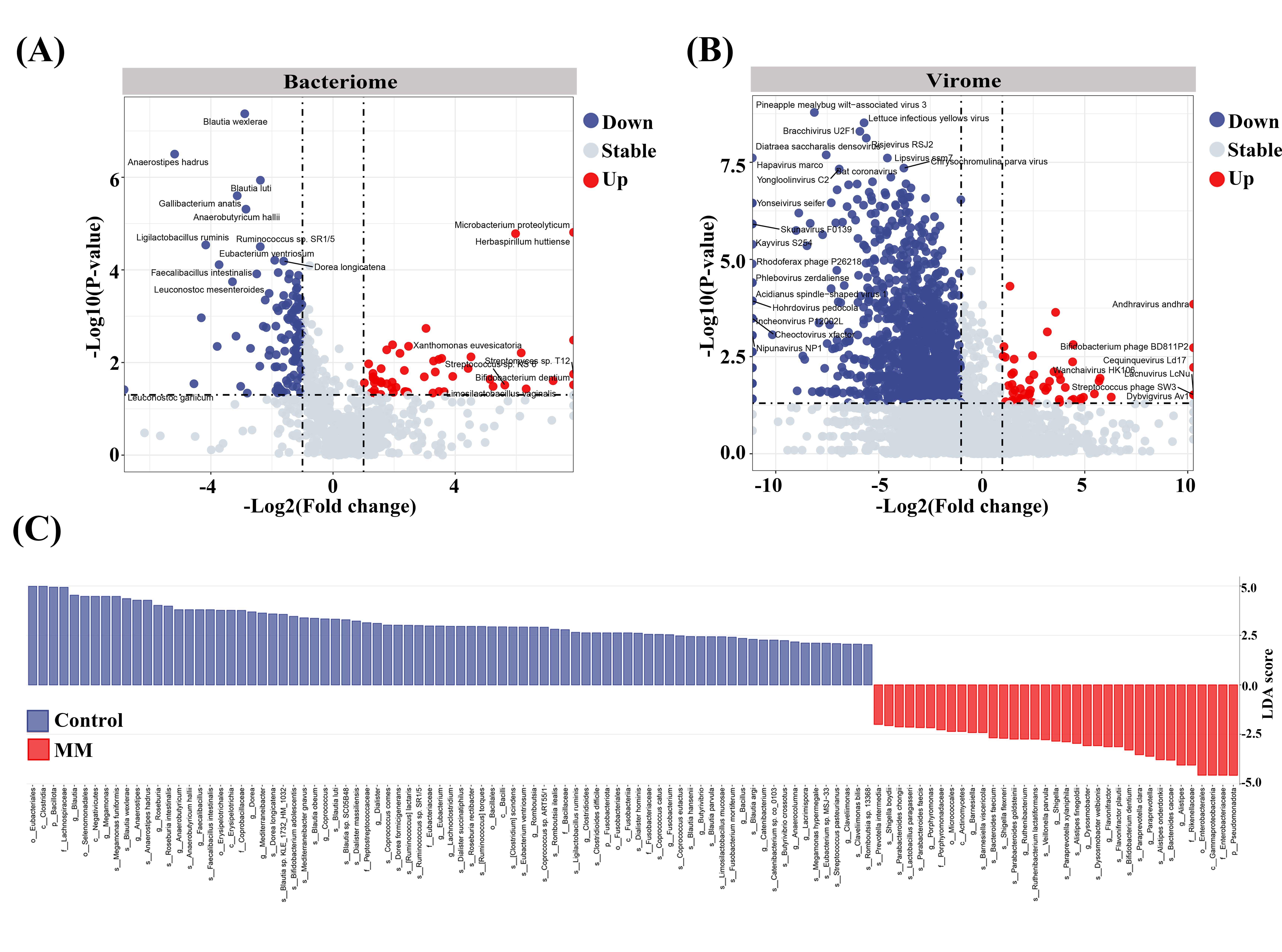

Supplement: SUPPLEMENTARY FIGURE S2 — (A,B) Volcano plot showing correlations between p-values and relative intensities (fold change, FC) for representative gut bacteria and viruses in Control and MM. Red and blue dots indicate significant differences. (C) Histogram of LDA scores computed for differentially abundant taxa between Control and MM groups. [file Image_2.tif]

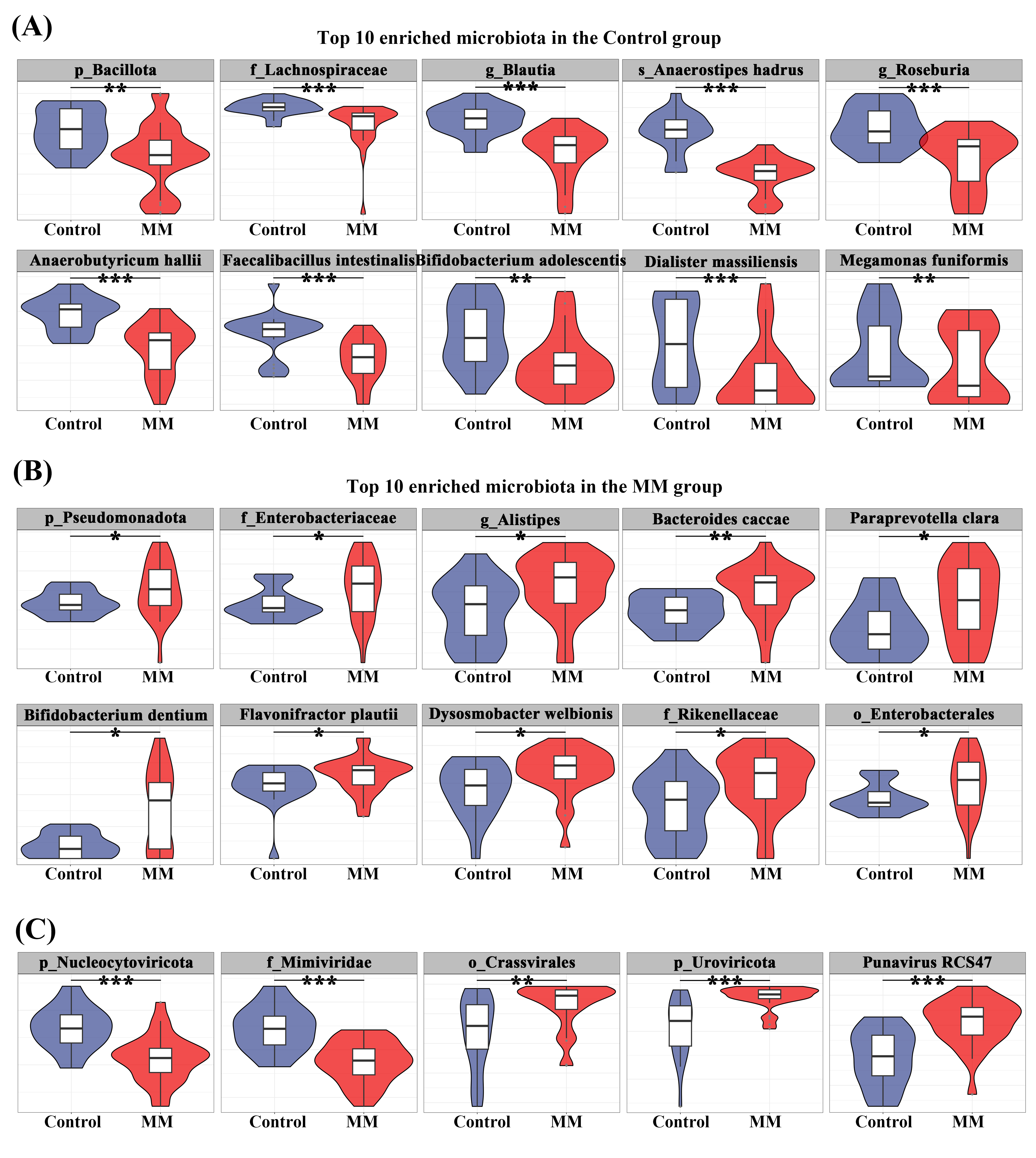

Supplement: SUPPLEMENTARY FIGURE S3 — (A,B) Relative abundance of the top 10 significant differential bacteria compared between Control and MM groups using the Metastats test. (C) Relative abundance of the top five significant differential viruses was compared between Control and MM groups using the Metastats test. [file Image_3.tif]

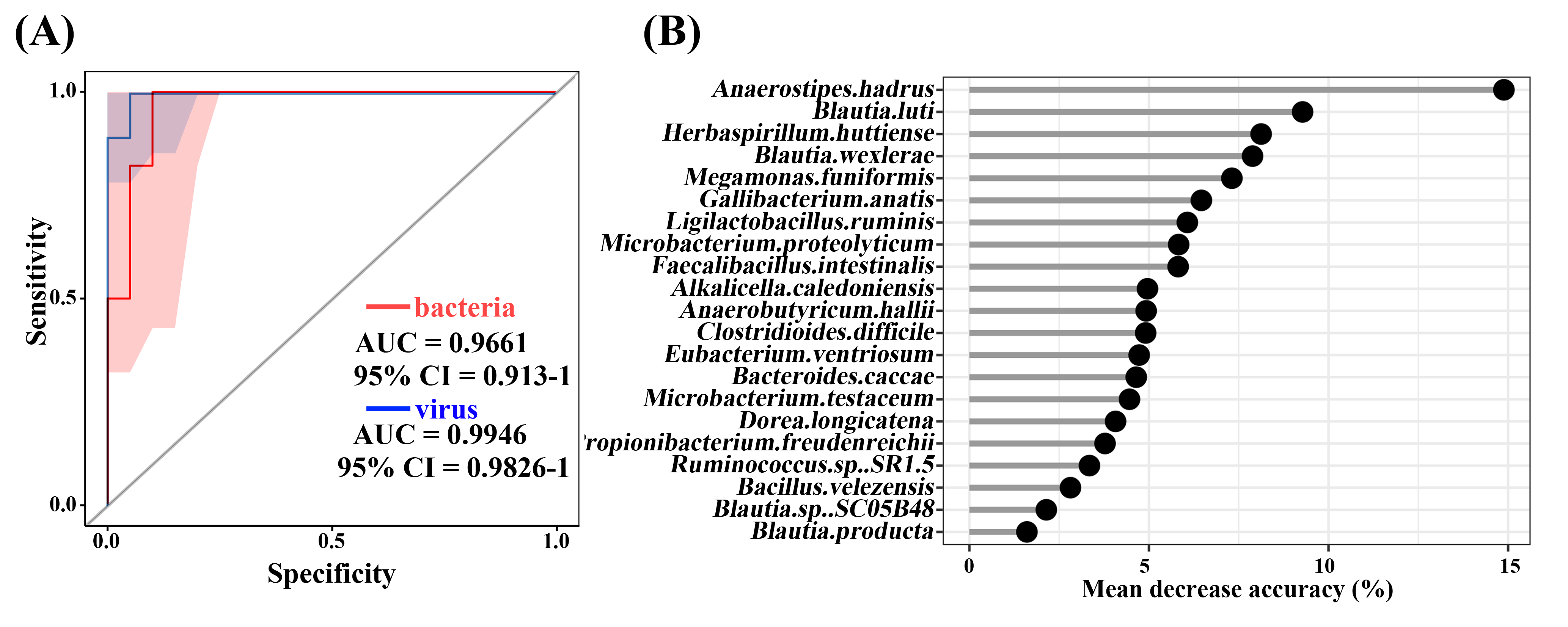

Supplement: SUPPLEMENTARY FIGURE S4 — Classification of MM status by the abundances of gut signatures (A) ROC analysis for classifying of MM status using gut bacterial and viral signatures. (B) Random forest analysis showing the importance scores for bacteria between Control and MM groups. [file Image_4.tif]

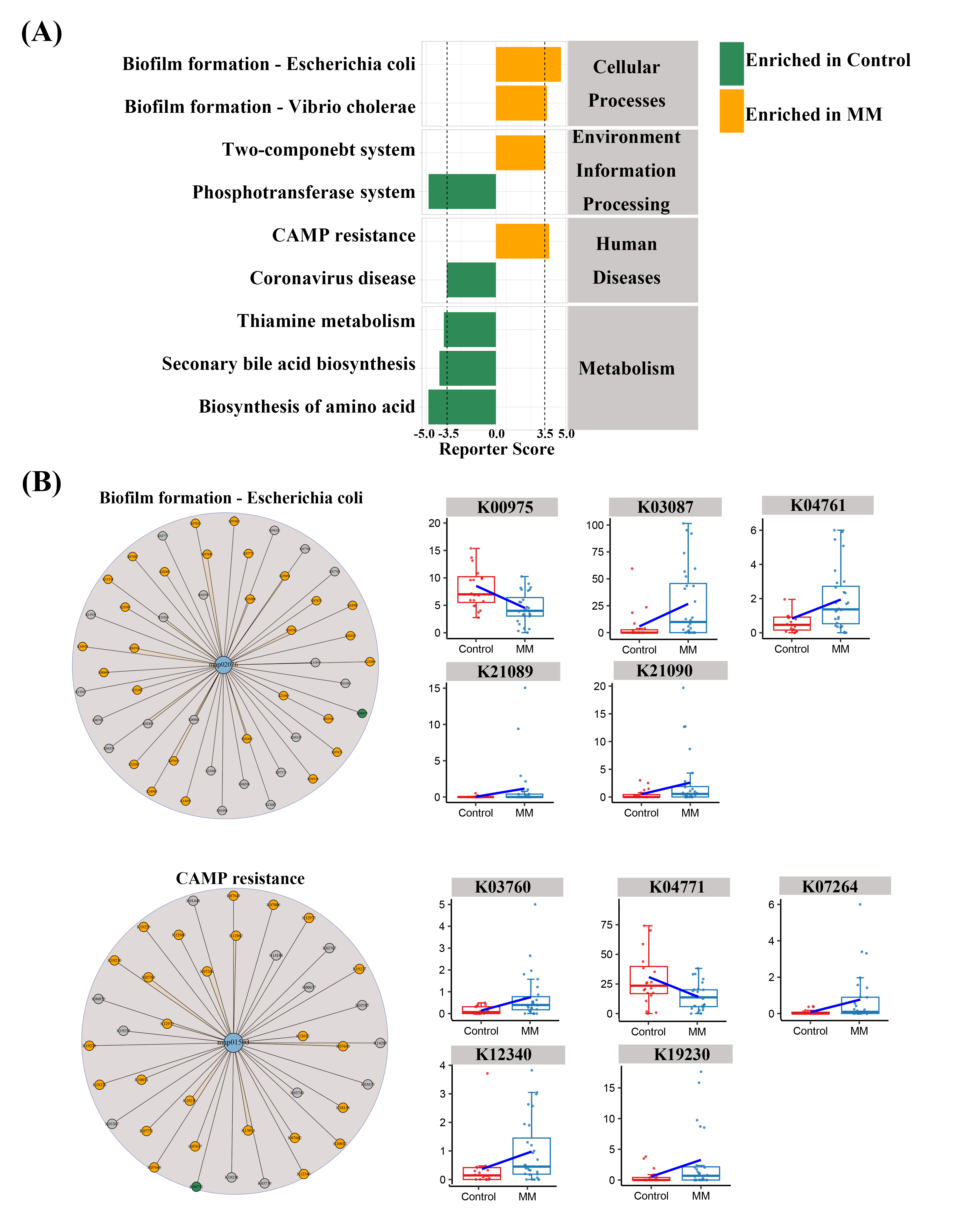

Supplement: SUPPLEMENTARY FIGURE S5 — Microbial functional alterations among MM patients. (A) Differentially enriched KEGG orthology (KO) genes between MM patients and controls. (B) Kyoto Encyclopedia of Genes and Genomes (KEGG) orthology (KO) genes associated with Escherichia coli and CAMP resistance (m02026 and m01503) are shown (left). Box plots show the relative abundance of the five most enriched KO genes in MM (right). [file Image_5.tif]

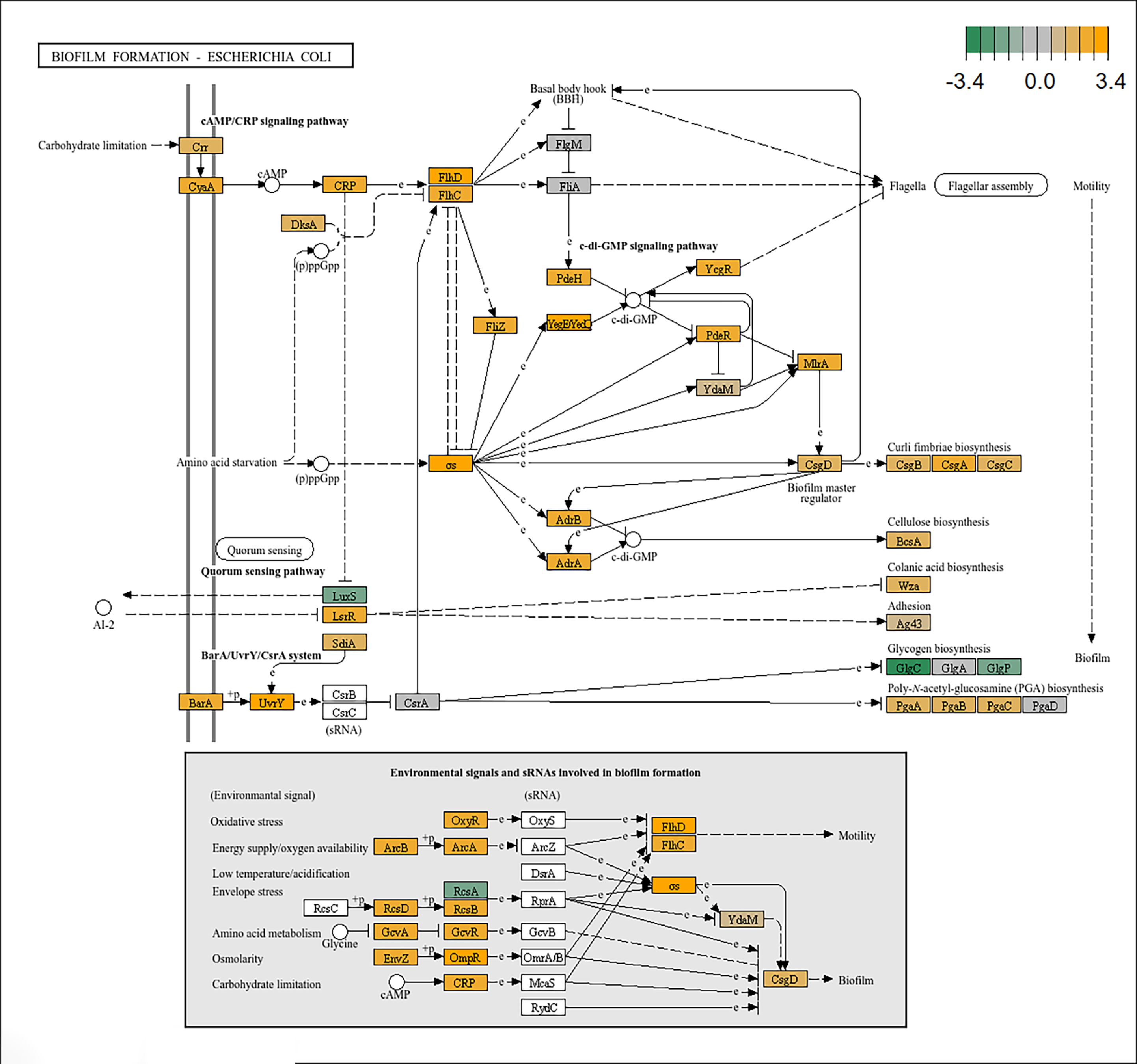

Supplement: SUPPLEMENTARY FIGURE S6 — KEGG pathway diagram of biofilm formation in Escherichia coli. [file Image_6.tif]

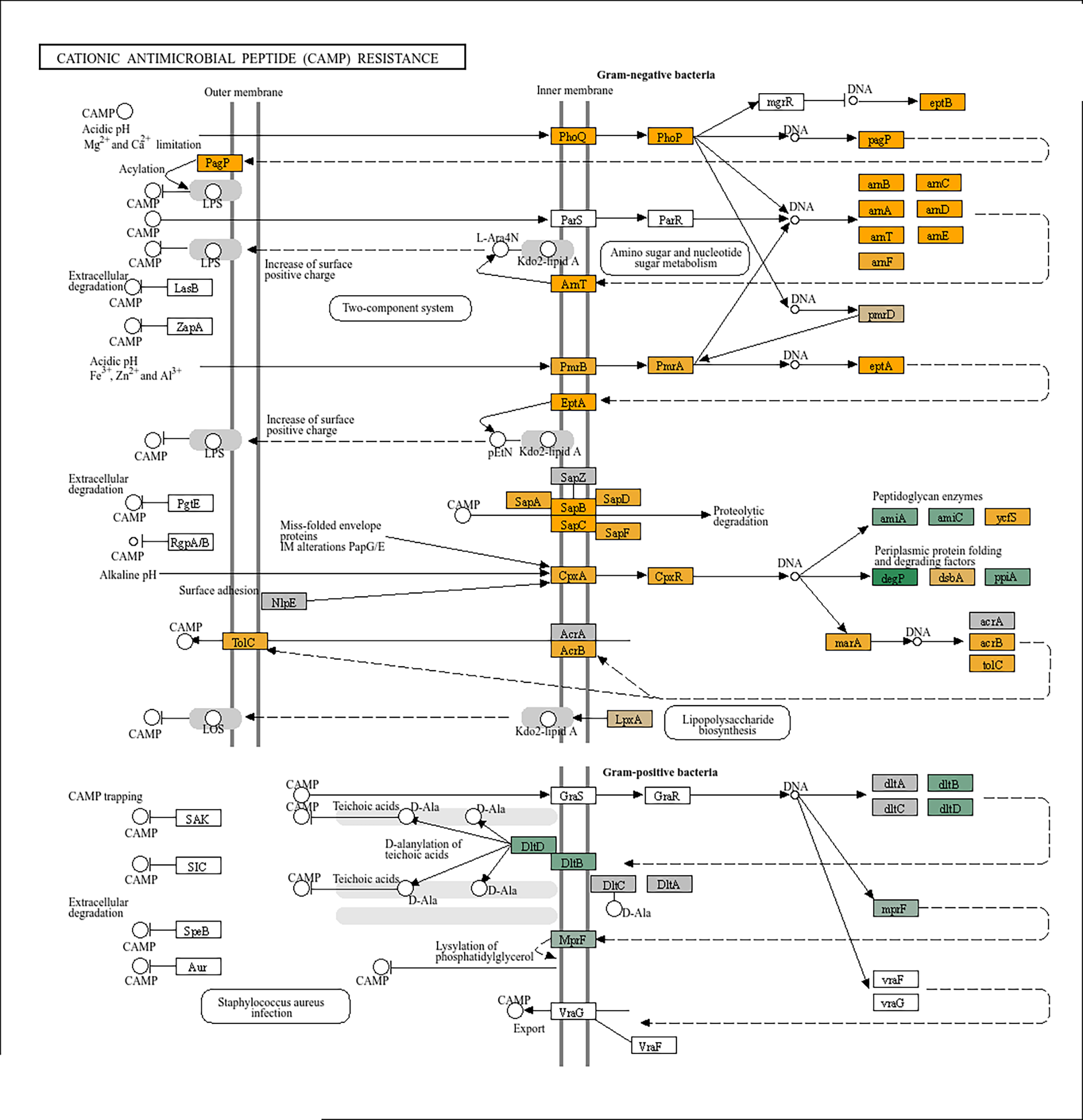

Supplement: SUPPLEMENTARY FIGURE S7 — KEGG pathway diagram of cationic antimicrobial peptide (CAMP) resistance. [file Image_7.tif]
