# Supplementary material for: Multi-kingdom gut microbiota analysis identifies bacterial-viral association in multiple myeloma
Source: Front Microbiol. 2026 May 29;17:1798330. doi: 10.3389/fmicb.2026.1798330 (PMC13262065; doi:10.3389/fmicb.2026.1798330)
Supplement: Supplementary file 8 [file Table_1.DOCX]

**Table S1 clinical characteristics of patients with MM**

| **Patients' characteristics** | **All patients (n=28; n/N*100%)** |
| --- | --- |
| WBC (×10⁹/L) | 8.25 |
| Hemoglobin (g/L) | 96.77 |
| Platelet (×10⁹/L) | 171.74 |
| Heavy chain (g/L) | 45.49 |
| β2-microglobulin (mg/L) | 10.27 |
| Calcium (mmol/L) | 2.29 |
| Creatinine (μmol/L) | 172.39 |
| Albumin (g/L) | 36.17 |
| M protein (g/L) | 30.51 |
| Serum κ chain (mg/L) | 3050.84 |
| Serum λ chain (mg/L) | 1742.31 |
| Serum κ/λ ratio | 327.81 |
| **Subtype of MM** |  |
| IgG | 17/28 (60.7%) |
| IgA | 9/28 (32.1%) |
| IgD | 0/28 (0.0%) |
| IgM | 1/28 (3.5%) |
| Nonsecretory/Other | 4/28 (14.3%) |
| **ISS staging** |  |
| Stage I | 0/28 (0.0%) |
| Stage II | 3/28 (10.7%) |
| Stage III | 14/28 (50.0%) |
